# Retraction Note To: The Association of Palmitoylethanolamide with Luteolin Decreases Autophagy in Spinal Cord Injury

**DOI:** 10.1007/s12035-024-04100-z

**Published:** 2024-03-12

**Authors:** Rosalba Siracusa, Irene Paterniti, Giuseppe Bruschetta, Marika Cordaro, Daniela Impellizzeri, Rosalia Crupi, Salvatore Cuzzocrea, Emanuela Esposito

**Affiliations:** 1 Department of Biological and Environmental Sciences, University of Messina, Viale Ferdinando Stagno D’Alcontres, 31, Messina, 98166 Italy; 2 Department of Pharmacological and Physiological Science, Saint Louis University School of Medicine, 1402 South Grand Blvd, St Louis, MO 63104 USA

**Retraction Note to: Mol Neurobiol (2016) 53:3783–3792**

10.1007/s12035-015-9328-6.

The Editor-in-Chief has retracted this article. After publication, concerns were raised regarding the western blot β-actin blots presented in Figs. 2a and 5a, b and c. Specifically, lanes 1–4 in these blots appear highly similar to lanes 6–9.

The authors have provided an alternative image for these figures, but were unable to sufficiently explain the similarities of the indicated bands.

The Editor-in-Chief therefore no longer has confidence in the presented data.

Salvatore Cuzzocrea has not explicitly stated whether he agrees to this retraction. None of the other co-authors have responded to any correspondence from the publisher about this retraction.

